# Supplementary material for: Tissue geometry and mechanochemical feedback initiate rotational migration in Drosophila
Source: bioRxiv. 2025 Sep 4:2025.09.03.674060. Preprint. [Version 1] doi: 10.1101/2025.09.03.674060 (PMC12424739; doi:10.1101/2025.09.03.674060)
Supplement: 1 [file NIHPP2025.09.03.674060V1-supplement-1.pdf]

bioRxiv preprint doi: https://doi.org/10.1101/2025.09.03.674060; this version posted September 4, 2025. The copyright holder for this preprint (which was not certified by peer review) is the author/funder, who has granted bioRxiv a license to display the preprint in perpetuity. It is made available under aCC-BY 4.0 International license.

Supplementary information for Tissue geometry and mechanochemical feedback initiate rotational migration in *Drosophila*

Sierra Schwabach[1,*], Sreejith Santhosh[2,*], Audrey Miller Williams[1,4], Maureen Cetera[1,3,5], Mattia Serra[2,†], and Sally Horne-Badovinac[1,3,†]

[1]Department of Molecular Genetics and Cell Biology, The University of Chicago, Chicago, IL, USA
[2]Department of Physics, University of California, San Diego, CA, USA
[3]Committee on Development, Regeneration, and Stem Cell Biology, The University of Chicago, Chicago, IL, USA
[4]Current address: Department of Cell Biology, Duke University, Durham, NC, USA
[5]Current address: Department of Genetics, Cell Biology and Development, University of Minnesota, Minneapolis, MN, USA

Contents

Supplementary Figure 1 2

Supplementary Figure 2 3

S1 Mechanochemical model of delayed migration egg chamber 4
S1.1 Mechanics of the egg chamber . . . 4
S1.1.1 Elastic confinement due to basement membrane . . . 5
S1.2 Crawling force and cell polarity dynamics . . . 6
S1.2.1 Fat2 polarity dynamics . . . 6
S1.2.2 Crawling force dynamics . . . 6
S1.3 Nondimensional equations of motion and parameter analysis . . . 7
S1.3.1 Parameters τ_1, τ_2 and τ_3 . . . 7
S1.4 Numerical implementation . . . 7
S1.5 Spherical egg chamber . . . 8

S2 Mechanochemical model of stage 1 egg chamber 8
S2.1 Mechanics of stage 1 egg chambers . . . 8
S2.1.1 Non-dimensional equation of motion and parameter selection . . . 9

Movie legends 11

Table 1: Key resources table 12

Table 2: Experimental genotypes 15

*These authors contributed equally to this work
†For correspondence:mserra@ucsd.edu , shorne@uchicago.edu

bioRxiv preprint doi: https://doi.org/10.1101/2025.09.03.674060; this version posted September 4, 2025. The copyright holder for this preprint (which was not certified by peer review) is the author/funder, who has granted bioRxiv a license to display the preprint in perpetuity. It is made available under aCC-BY 4.0 International license.

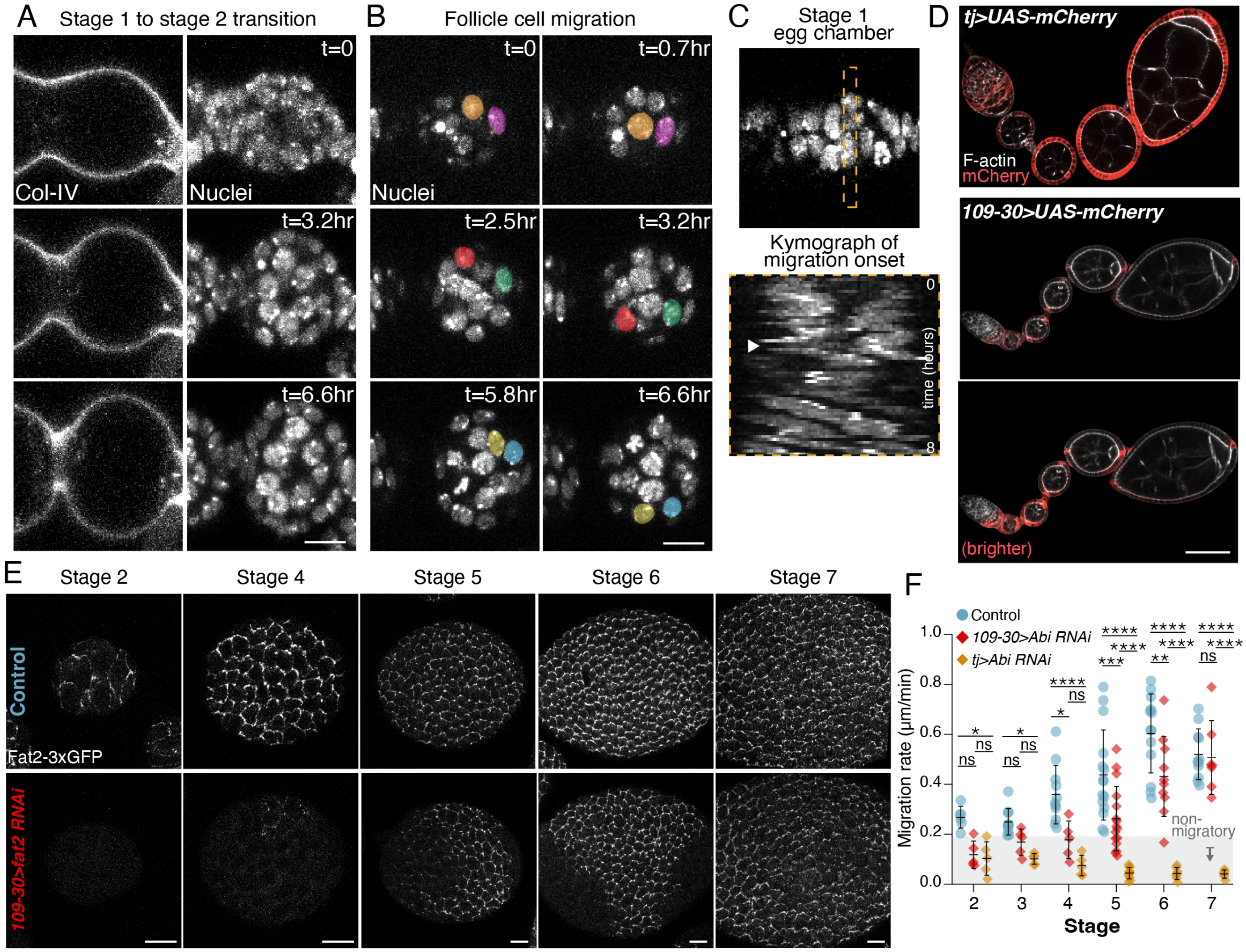

Figure S1: **Additional characterization of the onset of migration at stage 1, and delayed-migration onset, related to Figure 1 and 2. A)** Additional example of stage 1 to stage 2 transition. Movie stills of a transverse section through a stage 1 egg chamber. **B)** Movie stills focused on the follicular epithelium of the same egg chamber as in A. Two different cells are pseudocolored in each row of images to show cell movement. **C)** Additional example of migration onset (arrowhead) at stage 1. Scale bars, 10µm. **D)** Representative images showing the two Gal4 drivers used in this study driving *UAS-mCherry*. The *tj-Gal4* driver is expressed in the follicle cells throughout the stages when the egg chamber rotates, whereas the *109-30-Gal4* driver is primarily expressed in the earliest rotation stages. The first two images were taken with the same microscope settings. The fluorescence signal in the third image has been enhanced to better show the expression pattern. Scale bar, 50µm. **E)** Representative images of Fat2-3xGFP at the basal epithelial surface. *109-30>fat2-RNAi* eliminates Fat2 expression through stage 2, but Fat2 gradually reaches full expression by stage 7 as the RNAi stops being expressed. Scale bar, 10µm. **F)** Quantification of follicle cell migration rates over developmental time. *109-30>Abi RNAi* blocks migration at early stages but migration is indistinguishable from controls by stage 7. Each data point represents one egg chamber. Control values are repeated from Figure 2D. Two-way ANOVA with Tukey's multiple comparisons test; ns, $p > 0.05$, $*p < 0.05$, $**p < 0.01$, $***p < 0.001$, $****p < 0.0001$. In order on graph, $n = 6, 5, 5, 10, 5, 5, 11, 5, 5, 15, 16, 10, 13, 10, 7, 11, 7, 5$. Bars represent mean ± SD.

bioRxiv preprint doi: https://doi.org/10.1101/2025.09.03.674060; this version posted September 4, 2025. The copyright holder for this preprint (which was not certified by peer review) is the author/funder, who has granted bioRxiv a license to display the preprint in perpetuity. It is made available under aCC-BY 4.0 International license.

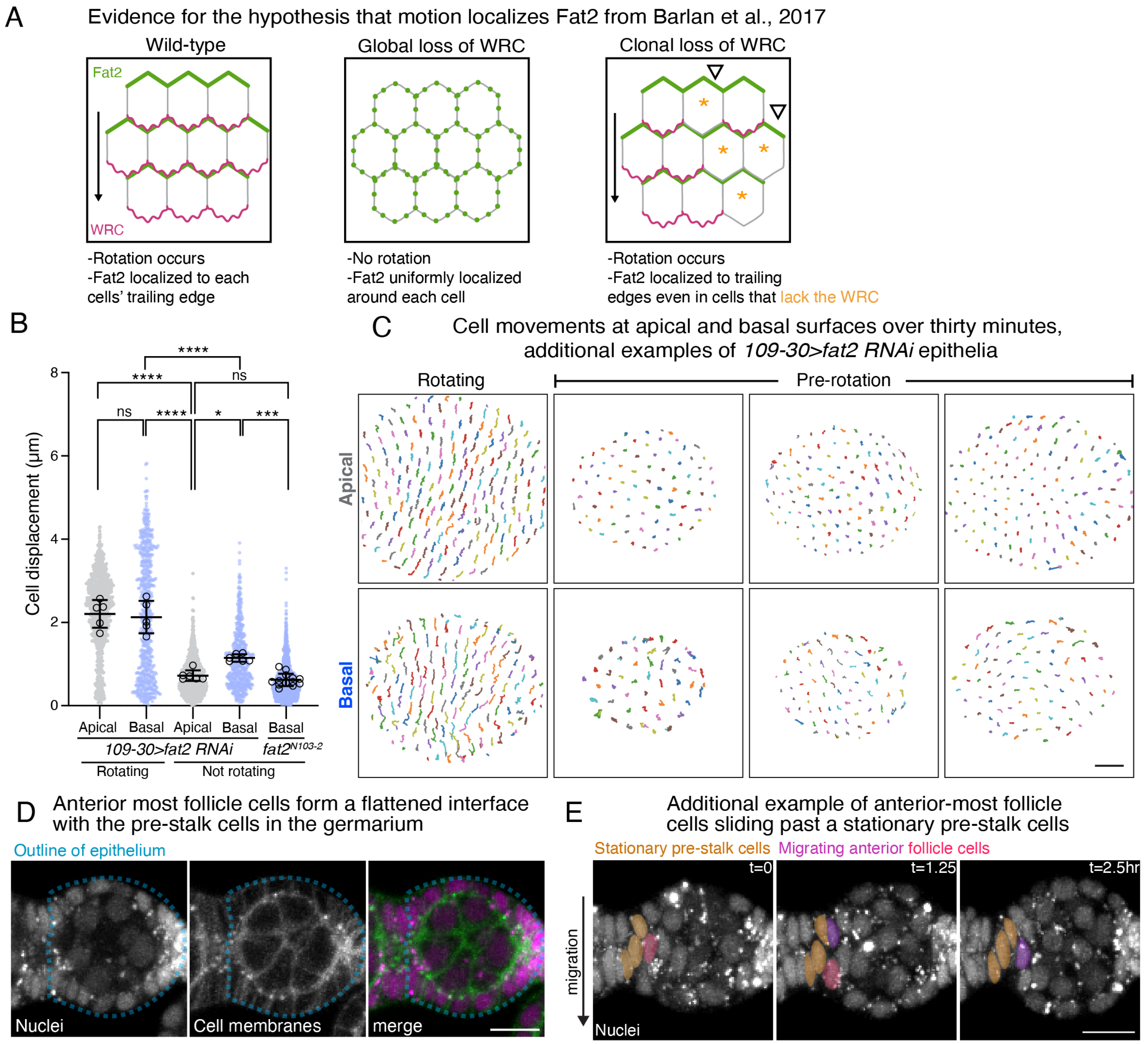

Figure S2: **Relationship between Fat2 polarity and global rotation; additional characterization of cell movement at apical and basal surface planes; additional example of follicle cell/pre-stalk interaction. Related to Figures 3, 4 and 6. A)** Illustration of the experiment from Barlan et al., 2017 that first suggested that the localization of Fat2 to the trailing edge of each follicle cells depends on tissue motion. **B)** Quantification of cell displacement at different imaging planes. Closed dots represent individual cells across multiple egg chambers, black open circles represent the average value for each egg chamber. For egg chamber averages: Ordinary one-way ANOVA with Tukey's multiple comparisons test; ns, $p > 0.05$, $^{*}p < 0.05$, $^{***}p < 0.001$, $^{****}p < 0.0001$. In order on graph, $n = 5, 5, 6, 6, 13$. Bars represent mean ± SD. **C)** Additional examples of cell centroid tracks over 30 minutes at apical and basal surface planes in rotating or not-rotating *109-30>fat2-RNAi* egg chambers. **D)** Transverse section of the stage 1 egg chamber shown in Fig. 6B, used to generate the diagram in Fig. 6A, highlighting the shape of the follicular epithelium in a rotating egg chamber at stage 1. Cell membranes were visualized using CellMask. **E)** Movie stills of maximum intensity projections of nuclei generated from a 2.5 hour movie. Stationary pre-stalk cell nuclei are pseudocolored gold, rotating pre-polar and follicle cell nuclei are pseudocolored magenta and blue, respectively. Scale bars = 10µm

bioRxiv preprint doi: https://doi.org/10.1101/2025.09.03.674060; this version posted September 4, 2025. The copyright holder for this preprint (which was not certified by peer review) is the author/funder, who has granted bioRxiv a license to display the preprint in perpetuity. It is made available under aCC-BY 4.0 International license.

S1 Mechanochemical model of delayed migration egg chamber

S1.1 Mechanics of the egg chamber

We model the egg chamber as a rigid body, as there are negligible neighbor exchanges between follicle cells, and these cells tightly adhere to the germ cells. Egg chambers have an ellipsoidal geometry and are slightly elongated along the AP axis at stages 5-6, when the delayed onset of rotation occurs. We parametrize the egg chamber (i.e. a surface $\mathcal{M}$, Fig S3A) as

$$\mathbf{r}(\theta,\phi) = \{a\cos\phi\sin\theta, a\sin\phi\sin\theta, ae\cos\theta\};\ \theta \in [0,\pi] \text{ and } \phi \in [0,2\pi), \tag{S1}$$

where a is the radius of the equatorial cross-section and e is the eccentricity (Fig. S3B). At each point on the egg chamber $\mathbf{r}(\theta,\phi)$, the tangent vector basis $({}_1\zeta, {}_2\zeta)$ and the surface element $\mathbf{dA}$ (Fig. S3A) are given by

$$\begin{cases} {}_1\zeta = \{\frac{\cos\theta\cos\phi}{\sqrt{\cos\theta^2+e^2\sin\theta^2}}, \frac{\cos\theta\sin\phi}{\sqrt{\cos\theta^2+e^2\sin\theta^2}}, -\frac{e\sin\theta}{\sqrt{\cos\theta^2+e^2\sin\theta^2}}\},\quad {}_2\zeta = \{-\sin\phi, \cos\phi, 0\} \\ \mathbf{dA} = g(\theta,\phi)\hat{\mathbf{n}}(\theta,\phi)d\theta d\phi,\quad g(\theta,\phi) = a^2\sqrt{\frac{1+e^2-(-1+e^2)\cos 2\theta}{2}}\sin\theta, \end{cases} \tag{S2}$$

where $\hat{\mathbf{n}}(\theta,\phi)$ is the outward normal vector. To describe the dynamics, we define an inertial (i.e. fixed) frame $\mathcal{E} : \{\hat{\mathbf{e_1}}, \hat{\mathbf{e_2}}, \hat{\mathbf{e_3}}\}$ and a body-frame $\mathcal{B} : \{\hat{\mathbf{d_1}}, \hat{\mathbf{d_2}}, \hat{\mathbf{d_3}}\}$ that rotates with the egg chamber and moves with its center of mass, with $\hat{\mathbf{d_3}}$ oriented along the long axis (Fig. S3B). The motion of a rigid body in 3D can be described by its center of mass velocity $\mathbf{v}$ and its angular velocity ω. The dynamics of the egg chamber given external forces and torques obeys linear momentum and angular momentum principles in the over-damped limit, which in the body-frame $\mathcal{B}$ [1], read

$$\mathbf{F} = \mathbf{0}, \mathbf{\Gamma} = \mathbf{0}, \tag{S3}$$

where $\mathbf{F}, \mathbf{\Gamma}$ are the total applied force and torque, which we model below.

Tangential drag force at the basement membrane. The basement membrane encases the egg chamber and resists its relative tangential motion via viscous drag due to remodeling of integrin-based adhesion complexes (Fig S3B). The basement membrane responds elastically to motion normal to the surface, which we model and discuss the consequences in Sec. S1.1.1. We model the tangential surface drag force density as

$$\mathbf{f}(\theta,\phi) = -\mu_b \mathbf{v}_{\parallel}(\theta,\phi), \tag{S4}$$

where μ_b is the viscous drag coefficient and $\mathbf{v}_{\parallel}$ is the component of the velocity tangential to the surface at (θ,ϕ). The velocity $\mathbf{v}(\theta,\phi)$ of a surface element is given by

$$\mathbf{v}(\theta,\phi) = \mathbf{v} + \omega \times \mathbf{r}(\theta,\phi), \tag{S5}$$

where $\mathbf{r}(\theta,\phi)$ is the position vector of the surface element with respect to the center of mass of the egg chamber. The total force and torque generated by the local viscous drag forces given in Eq. (S4) are obtained by integrating over the entire surface of the egg chamber (Eq. (S1)). Given that $e \approx 1.2$ for a stage 5 egg chamber we compute the total viscous friction force $\mathbf{F_f}$ and torque $\mathbf{\Gamma_f}$ (expressed in $\mathcal{B}$) by expanding about $e = 1$ in linear order, which gives

$$\begin{cases} \mathbf{F_f} = \int_{\mathcal{M}} \mathbf{f}(\theta,\phi)g(\theta,\phi)d\theta d\phi = -\frac{8}{3}a^2\pi\mu_b\mathbf{v} - \frac{4}{15}a^2\pi\mu_b(e-1)\begin{bmatrix} 2v_1 \\ 2v_2 \\ 11v_3 \end{bmatrix}, \\ \mathbf{\Gamma_f} = \int_{\mathcal{M}} \mathbf{r}(\theta,\phi) \times \mathbf{f}(\theta,\phi)g(\theta,\phi)d\theta d\phi = -\frac{8}{3}\mu_b a^4\pi\omega - \frac{2}{15}\mu_b a^4\pi(e-1)\begin{bmatrix} 31\omega_1 \\ 31\omega_2 \\ 13\omega_3 \end{bmatrix}, \end{cases} \tag{S6}$$

where $\mathbf{v} = [v_1, v_2, v_3]$ and $\omega = [\omega_1, \omega_2, \omega_3]$ are expressed in the body-frame $\mathcal{B}$.

Protrusive crawling force. The follicle cells use cryptic lamellipodia at their basal surfaces to crawl on the basement membrane [2]. We model these active point forces $\mathbf{b^i}$ acting at the cell-centroid $\mathbf{r^i} \in \mathcal{M}$ (Fig S3C) on the

bioRxiv preprint doi: https://doi.org/10.1101/2025.09.03.674060; this version posted September 4, 2025. The copyright holder for this preprint (which was not certified by peer review) is the author/funder, who has granted bioRxiv a license to display the preprint in perpetuity. It is made available under aCC-BY 4.0 International license.

basal surface of cell i. Therefore, the total crawling (or active) force $\mathbf{F}_a$ and torque $\mathbf{\Gamma}_a$ exerted by the basal surface protrusions are given in the body frame by

$$\mathbf{F}_a = \sum_{i=1}^{N} \mathbf{b^i}, \quad \mathbf{\Gamma}_a = \sum_{i=1}^{N} \mathbf{r^i} \times \mathbf{b^i}. \tag{S7}$$

S1.1.1 Elastic confinement due to basement membrane

The basement membrane elastically resists motion perpendicular to the egg chamber-basement membrane interface. The elastic surface force density is given by

$$\mathbf{f}(\theta, \phi) = -K_B(\delta r_\perp(\theta, \phi)/h)\hat{\mathbf{n}}(\theta, \phi) \tag{S8}$$

where K_B, h are the stiffness and thickness of the basement membrane, and $\delta r_\perp(\theta, \phi)$ is the displacement of the area element normal to the interface. For an egg chamber with the center of mass displaced by $\delta \mathbf{r}^C = [\delta r_1^C, \delta r_2^C, \delta r_3^C]$ in the body-frame $\mathcal{B}$ and body-frame orientation given by rotation matrix $\mathbf{A}$,

$$\delta r_\perp(\theta, \phi) = (\delta \mathbf{r}^C + (\mathbf{A} - \mathbb{I})\mathbf{r}(\theta, \phi)) \cdot \hat{\mathbf{n}}(\theta, \phi). \tag{S9}$$

The rotation matrix $\mathbf{A}$ gives the orientation of the body-frame $\mathcal{B}$ with respect to the inertial-frame $\mathcal{E}$. The orientational rest-configuration of the egg chamber is assumed to be $\mathbf{A} = \mathbb{I}$ and we analyze small deviations $\delta\theta$ of the egg chamber AP axis away from $\hat{\mathbf{e_3}}$ (Fig S3D-E). Integrating over the egg chamber, we obtain the total force and torque to linear order in e represented in the $\mathcal{B}$ frame:

$$\begin{cases} \mathbf{F}_e = \int_{\mathcal{M}} \mathbf{f}(\theta, \phi) g(\theta, \phi) d\theta d\phi = -\frac{4}{3h} a^2 \pi K_B \delta \mathbf{r}^C - \frac{8}{15h} a^2 \pi K_B (e-1) \begin{bmatrix} 3\delta r_1^C \\ 3\delta r_2^C \\ \delta r_3^C \end{bmatrix}, \\ \mathbf{\Gamma_e} = \int_{\mathcal{M}} \mathbf{r}(\theta, \phi) \times \mathbf{f}(\theta, \phi) g(\theta, \phi) d\theta d\phi = -\frac{8}{15h} K_B a^4 \pi (e-1) \begin{bmatrix} A_{23} + A_{32} \\ A_{13} + A_{31} \\ 0 \end{bmatrix}. \end{cases} \tag{S10}$$

Active crawling forces drive the rigid rotation. An order of magnitude estimate of the active force $\mathbf{F}_a$ and torque $\mathbf{\Gamma}_a$ is given by

$$|\mathbf{F}_a| = N b_c, |\mathbf{\Gamma}_a| = N b_c a, \tag{S11}$$

where b_c is the characteristic crawling force of one cell. $b_c = \sigma_a A_c$, where σ_a is the mean traction stress generated by a crawling cell and A_c is the cell area. There are currently no available experimental measurements of σ_a in the *Drosophila* egg chamber. Instead, we use an estimate of traction stresses ≈ 10 Pa measured from collectively migrating epithelial cells in another *in vivo* system [3]. Equating the magnitude of active crawling and elastic response force (torque), we can estimate the theoretical maximum translation (rotational motion) of the egg chamber. Using the values of $K_B \approx 50$kPa, $h \approx 90nm$, $a \approx 25\mu m$, $e \approx 1.2$ [4–7] we get,

$$|\delta \mathbf{r}^C| \approx 5.4 \times 10^{-2} \text{ nm}, A_{23} + A_{32} \text{ and } A_{31} + A_{13} \approx 2.7 \times 10^{-5}. \tag{S12}$$

Therefore, since the motion of the center of mass is significantly smaller than the dimensions of the egg chamber ($|\delta \mathbf{r}^C| << a$), we assume that the center of mass remains stationary for all our simulations. The entries $A_{23}, A_{32}, A_{31}, A_{13}$ of the orientation matrix $\mathbf{A}$ quantifies the deviation of the AP axis ($\hat{\mathbf{d_3}}$) from the rest orientation ($\hat{\mathbf{e_3}}$). Since Eq. (S12) holds for any rotation of the egg chamber about an axis in the $\{\hat{\mathbf{d_1}}, \hat{\mathbf{d_2}}\}$ plane, we can independently infer that $A_{32}, A_{23}, A_{31}, A_{13} \approx 2.7 \times 10^{-5}$. This demonstrates that rotation of the egg chamber is confined to its long axis, consistent with experimental observations [8]. This constraint on rotational axis does not hold for spherical egg chambers ($e \to 1$), which can rotate along any axis (See Sec. S1.5).

bioRxiv preprint doi: https://doi.org/10.1101/2025.09.03.674060; this version posted September 4, 2025. The copyright holder for this preprint (which was not certified by peer review) is the author/funder, who has granted bioRxiv a license to display the preprint in perpetuity. It is made available under aCC-BY 4.0 International license.

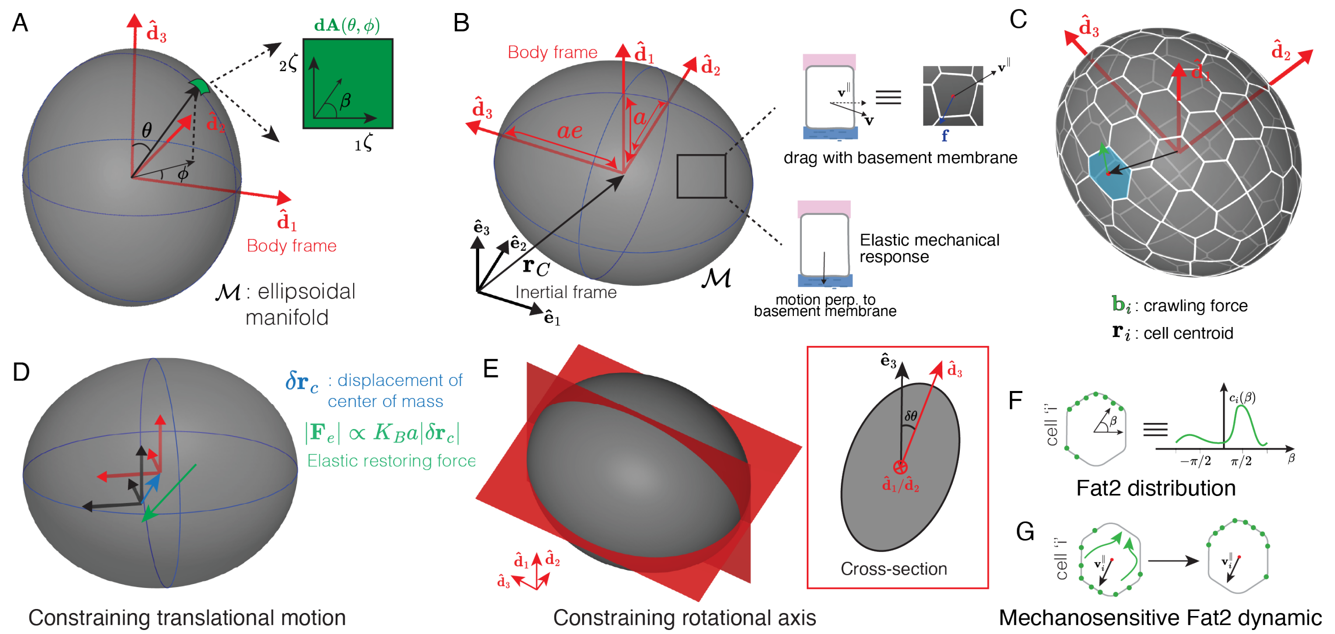

Figure S3: **Biophysical model of egg chambers during delayed migration onset.** (a) Egg chamber geometry is described using an ellipsoidal manifold $\mathcal{M}$ parameterized using angles (θ, ϕ) in the body frame $\mathcal{B} : \{\hat{\mathbf{d}}_1, \hat{\mathbf{d}}_2, \hat{\mathbf{d}}_3\}$. The area element $\mathbf{dA}$ at (θ, ϕ) has tangent bases vectors $\{{}_1\zeta, {}_2\zeta\}$ and β is the angular coordinate on the tangent plane. (b) The dynamics can be described both in the inertial frame $\mathcal{E}$ and the body frame $\mathcal{B}$. The basement membrane provides viscous (elastic) resistance force to motion tangential (perpendicular) to it. (c) Follicle cell positions described in the body frame. (d-e) Elastic response of the basement membrane to motion of the egg chamber normal to the interface constrains translational motion (d) and rotational axis (e). (f) The Fat2 concentration for cell i is described using $c^i(\beta)$. (g) The Fat2 concentration is mechanosensitive.

S1.2 Crawling force and cell polarity dynamics

S1.2.1 Fat2 polarity dynamics

We model the Fat2 distribution for cell i as $c^i(\beta, t)$, where β is the angular coordinate in the tangent space of $\mathcal{M}$ at the cell-centroid position $\mathbf{r}^i$ (Fig. S3F). Previous work [9] has demonstrated that Fat2 localizes to cell's trailing edges, and also suggests that tissue rotation may play a role in localizing Fat2. Therefore, we construct a minimal model of mechanosensitive Fat2 dynamics as

$$\frac{\partial c^i(\beta)}{\partial t} = -\epsilon_0(\epsilon_1 \mathbf{v}^i \cdot \mathbf{m}(\beta, \mathbf{r}^\mathbf{i}) + c^i(\beta))c^i(\beta), \quad \mathbf{m}(\beta, \mathbf{r}^\mathbf{i}) = \cos(\beta(t))_1\zeta(\mathbf{r}^i) + \sin(\beta(t))_2\zeta(\mathbf{r}^i), \tag{S13}$$

where $\mathbf{v}^i = \mathbf{v} + \omega \times \mathbf{r}^i$ is velocity of cell i, ϵ_0 is a characteristic rate constant for the dynamics of Fat2 polarity and ϵ_1 sets the strength of coupling between migration and Fat2 polarity. Intuitively, Eq. (S13) models an increase in Fat2 polarization to the cell edge in the direction opposite of motion (Fig. S3G).

S1.2.2 Crawling force dynamics

Fat2 acts at cells' trailing edges to create a stable protrusive domain in the leading edge of the neighboring cell behind [9]. Thus we assume the direction of the crawling force $\mathbf{b}^i$ to be influenced by Fat2 polarity. For simplicity, we model the activity of Fat2 as cell-autonomous and the crawling force $\mathbf{b}^i$ as

$$\partial_t \mathbf{b}^\mathbf{i} = \omega \times \mathbf{b}^\mathbf{i} - \frac{1}{\gamma}(\zeta(t) + \mathbf{b}^\mathbf{i}), \quad \zeta(t) = b_c(\cos(\beta(t))_1\zeta(\mathbf{r}^i) + \sin(\beta(t))_2\zeta(\mathbf{r}^i)), \tag{S14}$$

where $\beta(t)$ is sampled from the probability distribution $P_i(\beta) \propto c_i(\beta)$, b_c is the characteristic scale of the force generated by cell protrusions and γ is the timescale taken by the protrusion to respond to changes in Fat2 polarity

bioRxiv preprint doi: https://doi.org/10.1101/2025.09.03.674060; this version posted September 4, 2025. The copyright holder for this preprint (which was not certified by peer review) is the author/funder, who has granted bioRxiv a license to display the preprint in perpetuity. It is made available under aCC-BY 4.0 International license.

encoded in $\zeta(t)$. The term $\omega \times \mathbf{b_i}$ arises from differentiation in rotating frames and maintains the consistency of the protrusion direction during rigid rotation of the egg chamber.

S1.3 Nondimensional equations of motion and parameter analysis

Equations (S6-S7,S13-S14) constitute a mechanochemical model of the egg chamber. We nondimensionalize the variables using the transformations $t \to t_c t$, $\omega \to \omega_c \omega$, $\mathbf{r}_i \to a\mathbf{r}_i$, $\mathbf{v} \to \omega_c a\mathbf{v}$, $\mathbf{b_i} \to b_c \mathbf{b}_i$, $c_i \to c_c c_i$ and $\zeta \to b_c \zeta$; where t_c, ω_c,v_c, b_c, c_c are the characteristic time-scale of rotation initiation, angular-speed of egg chamber, force scale of protrusions and characteristic concentration scale of Fat2. Rotation initiation occurs over a time-scale $t_c \approx 1hour$. The radius of the egg chamber equatorial cross-section $a \approx 25\mu m$ during rotation initiation. The characteristic angular velocity $\omega_c \approx t_c^{-1}$ for an egg chamber during delayed rotation initiation. We further use the fact that after symmetry breaking, the egg chamber rotates in a single direction with an angular velocity $\omega \approx \omega_c$. In this regime, torque from crawling forces must balance torque from drag with the basement membrane, which, using the angular momentum balance, gives $b_c a N \approx \mu_b a^4 \omega_c$ (order of magnitude calculations), leading to $b_c \approx \frac{1}{N}\mu_b a^3 \omega_c$. Due to the strong elastic confinement from the basement membrane, the center of mass remains stationary and rotations are restricted to the long axis (See Sec. S1.1.1). Therefore, we obtain the non-dimensional equations of motion

$$\begin{cases} \Pi_\omega(e)\omega_3 = (\sum_{i=1}^{N} \mathbf{r}^i \times \mathbf{b}^i) \cdot \hat{\mathbf{d_3}} \text{ for } e > 1 \\ \partial_t \mathbf{b}^i = \omega \times \mathbf{b}^i - \tau_1(\zeta(t) + \mathbf{b}^i) \\ \partial_t c^i = -\tau_2(\tau_3 \mathbf{v}^i \cdot \mathbf{m}(\beta, \mathbf{r}^i) + c^i)c^i \end{cases}, \quad \begin{cases} \Pi_\omega(e) = N\pi(40 + 26(e-1))/15 \\ \tau_1 = t_c/\gamma, \tau_2 = \epsilon_0 t_c c_c, \tau_3 = \epsilon_1 a/t_c c_c \end{cases}. \tag{S15}$$

For simulations of the delayed onset of rotation, we use $e = 1.2$ and $N = 300$ consistent with experimental observations [7]. To compute the cell centroid locations $\mathbf{r}^i$ for a given number of follicle cells N, we initialized it by placing N equi-distant points on the surface of an ellipsoid.

S1.3.1 Parameters τ_1, τ_2 and τ_3

Rotation initiation occurs over a time-scale $t_c \approx 1hour$, and the time-scale over which protrusions respond to changes in Fat2 distribution is $\gamma \approx 3$ min [10]. Therefore, we set $\tau_1 = 20$. There is currently no reliable experimental estimate for (τ_2, τ_3). Therefore, we numerically explore the parameter space and observe that the egg chamber undergoes symmetry breaking for all positive values, but the time taken to break symmetry changes. We quantify this difference by estimating the time taken to reach 95 percent of the ensemble average of the steady state angular velocity $|\omega_3|^s$ after symmetry breaking (See SI Fig S4 A) for a parameter set. Increasing (τ_2, τ_3) decreases the time taken for the egg chamber to symmetry break (See SI Fig S4 B-C).

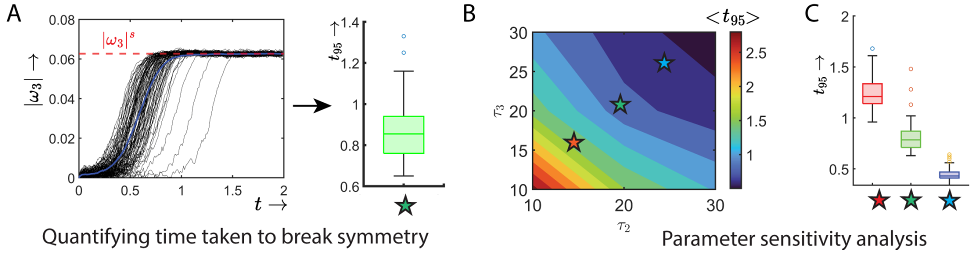

Figure S4: **Sensitivity analysis to changes in** (τ_2, τ_3)**.** (a) $N = 100$ runs of the theoretical model of a delayed egg chamber for the parameter values $(\tau_2, \tau_3) = (20, 20)$. (b-c) The ensemble average time taken $< t_{95} >$ to reach 95 percent of the steady-state symmetry broken angular velocity $|\omega_3|^s$ for different parameter sets.

S1.4 Numerical implementation

We simulate an egg chamber undergoing delayed rotation initiation by solving Eqns. (S15) using forward Euler integration method with time-step $dt = 10^{-2}$ in MATLAB. The evolution of the body frame is tracked using a rotation matrix $\mathbf{A}(t)$ which relates vectors in the inertial-frame $\mathcal{E}$ and body-frame $\mathcal{B}$ as

$$\mathbf{r}_E = A(t)\mathbf{r}_B, \tag{S16}$$

bioRxiv preprint doi: https://doi.org/10.1101/2025.09.03.674060; this version posted September 4, 2025. The copyright holder for this preprint (which was not certified by peer review) is the author/funder, who has granted bioRxiv a license to display the preprint in perpetuity. It is made available under aCC-BY 4.0 International license.

where $\mathbf{r}_E, \mathbf{r}_B$ are the coordinates of the same vector in the inertial and body frames. The rotation matrix $\mathbf{A}(t)$ evolves using the relation

$$\frac{d}{dt}\mathbf{A}(t) = \mathbf{\Omega A}, \quad \mathbf{\Omega} = \begin{pmatrix} 0 & -\omega_3 & \omega_2 \\ \omega_3 & 0 & -\omega_1 \\ -\omega_2 & \omega_1 & 0 \end{pmatrix}, \tag{S17}$$

where $\omega = [\omega_1, \omega_2, \omega_3]$ is the angular velocity of the egg chamber represented in the inertial frame. For an ellipsoidal ($e > 1$) egg chamber $\omega_2, \omega_1 = 0$ due to the strong confinement from the basement membrane (Sec S1.1.1).

S1.5 Spherical egg chamber

In contrast to an elongated egg chamber, the elastic confinement from the basement membrane does not constrain the rotational axis in a spherical egg chamber (Sec S1.1.1 for details). Therefore, using the same non-dimensionalization scheme as introduced in Sec. S1.3 the non-dimensional equation of motion for a spherical egg chamber is given by

$$\Pi_\omega(1)\omega = \sum_{i=1}^{N} \mathbf{r}^i \times \mathbf{b}^i. \tag{S18}$$

To simulate rotation initiation in a spherical egg chamber, we use Eq. (S18) for the rotational dynamics and the same equations for Fat2 concentration and crawling force as in the elongated egg chamber (Eq. (S15)). We simulate these equations with the same parameters τ_{1-3} as the delayed migration egg chamber, but with $N = 100$, as quantified for the stage 1 egg chamber. Starting from an isotropic initial condition ($c^i(\beta, 0) = 1/2\pi, \mathbf{b}^i(0) = \mathbf{0}$), the spherical egg chamber undergoes symmetry breaking (Fig S5A-C) but with a randomly oriented rotational axis (Fig S5D).

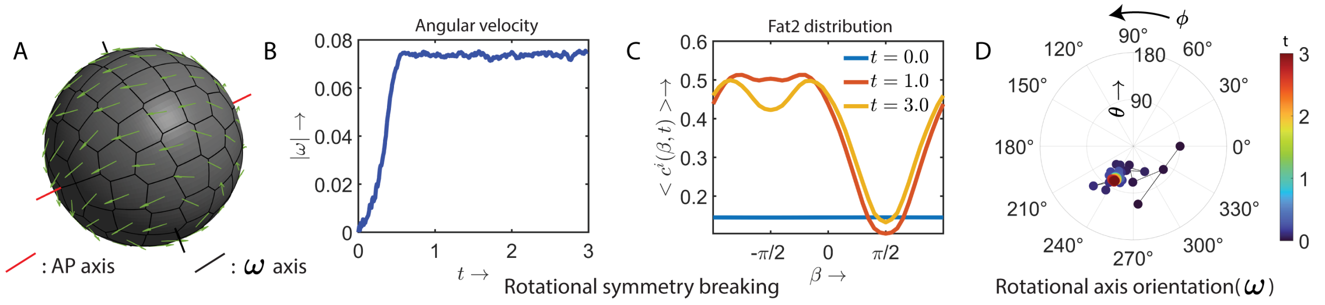

Figure S5: **Random rotational axis in spherical egg chambers.** (a) Rotation of a spherical egg chamber. (b-c) The egg chamber undergoes symmetry breaking, generating sustained rotations. (d) The orientation of the angular velocity ω described in the inertial frame $\mathcal{E}$ is quantified using spherical coordinates (θ, ϕ). The time-evolution of the quantities in a-d is given in Movie 10. We mark the future AP axis (red) for reference.

S2 Mechanochemical model of stage 1 egg chamber

S2.1 Mechanics of stage 1 egg chambers

The mechanics of stage 1 egg chambers is distinct from the later stages due to the contact of follicle cells with the pre-stalk cells (Fig. S6A). We model the mechanical interaction with the stationary pre-stalk cells to elucidate how the stage 1 egg chamber undergoes symmetry breaking. The stage 1 egg chamber is spherical ($e = 1$), but with a flat contact with the pre-stalk cells. The spatial extent of the contact region is quantified using the polar angle θ_s. We use a similar framework to the model introduced in Sec. S1.1, but accounting for the interaction with the pre-stalk cells. The strong confinement from the basement membrane, discussed in Sec. S1.1.1, holds even for the stage 1 egg chamber; therefore, the center of mass is fixed. We next discuss the changes in the external torques of this system compared to the delayed migration model.

bioRxiv preprint doi: https://doi.org/10.1101/2025.09.03.674060; this version posted September 4, 2025. The copyright holder for this preprint (which was not certified by peer review) is the author/funder, who has granted bioRxiv a license to display the preprint in perpetuity. It is made available under aCC-BY 4.0 International license.

Tangential drag force at the basement membrane. The tangential drag force is the same as for the egg chamber at the time of delayed-rotation onset Eq. (S4), but the surface of integration is given by $\mathcal{M}_b$ (Fig. S6B):

$$\mathbf{r}(\theta,\phi) = \{a\cos\phi\sin\theta, a\sin\phi\sin\theta, a\cos\theta\};\ \theta \in [\theta_s,\pi] \text{ and } \phi \in [0,2\pi). \tag{S19}$$

The total (friction) torque due to the drag with the basement membrane is given by

$$\mathbf{\Gamma_f} = \int_{\mathcal{M}_b} \mathbf{r}(\theta,\phi)\times\mathbf{f}(\theta,\phi)g(\theta,\phi)d\theta d\phi = -\frac{1}{12}\mu_b a^4\pi \begin{bmatrix}(16+15\cos\theta_s+\cos 3\theta_s)\omega_1\\(16+15\cos\theta_s+\cos 3\theta_s)\omega_2\\32\cos(\theta_s/2)^4(2-\cos\theta_s)\omega_3\end{bmatrix}. \tag{S20}$$

Tangential drag force at the pre-stalk interface. We assume a viscous drag between the stationary pre-stalk and the egg chamber for motion tangential to the interface. The surface drag force density is given by

$$\mathbf{f}(\eta,\rho) = -\mu_s\mathbf{v}_{\|}(\eta,\rho),\quad \mathbf{v}_{\|}(\eta,\rho) = \mathbf{v}(\eta,\rho) - (\mathbf{v}(\eta,\rho)\cdot\hat{\mathbf{d}}_3)\hat{\mathbf{d}}_3, \tag{S21}$$

where (η,ρ) is the polar parameterization of the pre-stalk interface (See Fig S6B), μ_s is the drag coefficient for the viscous interaction between the egg chamber and the pre-stalk, and $\mathbf{v}(\eta,\rho)$ is the velocity of the area element. The torque exerted by the viscous force given in Eq. (S21) is given by

$$\tau(\eta,\rho) = -\mathbf{r}(\eta,\rho)\times\mu_s\mathbf{v}_{\|}(\eta,\rho), \tag{S22}$$

where $\mathbf{r}(\eta,\rho)$ is the position vector of the area element at (η,ρ). Integrating Eq. (S22) over the egg chamber/pre-stalk interface $\mathcal{M}_s$, we get the corresponding total torque exerted by this viscous force:

$$\mathbf{\Gamma}_s = \int_{\mathcal{M}_s}\tau(\eta,\rho)dA = -\frac{1}{2}\mu_s a^4\sin(\theta_s)^2\pi\begin{bmatrix}2\cos(\theta_s)^2\omega_1\\2\cos(\theta_s)^2\omega_2\\\sin(\theta_s)^2\omega_3\end{bmatrix}. \tag{S23}$$

Elastic confinement at the pre-stalk interface. Motion of an area element $\mathbf{dA}$ at (η,ρ) on the stalk-egg chamber interface normal to it generates an elastic resisting force. The elastic surface force density is given by

$$\mathbf{f}_e(\eta,\rho) = -k_s r_\perp(\eta,\rho)\hat{\mathbf{e}}_3,\quad r_\perp(\eta,\rho) = (\omega\times\mathbf{r}(\eta,\rho))\cdot\hat{\mathbf{e}}_3 - R\cos\theta_s, \tag{S24}$$

where k_s is the stiffness of the pre-stalk cells and $r_\perp(\eta,\rho)$ is the displacement of the area element normal to the interface (See Fig. S6C). Similar to the tangential drag force given above, we compute the torque and integrate over the interface $\mathcal{M}_s$ to get the total elastic torque

$$\mathbf{\Gamma}_e = \frac{1}{8}k_s\pi a^4\sin(\theta_s)^2\begin{bmatrix}A_{32}(-4+3A_{33}+(-4+5A_{33})\cos 2\theta_s)\\A_{31}(4-3R_{33}+(4-5A_{33})\cos 2\theta_s)\\0\end{bmatrix}, \tag{S25}$$

where A_{ij} are the components of the rotation matrix that transforms vectors from the body frame $\mathcal{B}$ to $\mathcal{E}$.

S2.1.1 Non-dimensional equation of motion and parameter selection

We use the same non-dimensionalization scheme in Sec. S1.3. The Fat2 polarity and protrusion dynamics are as above, while the angular momentum balance is different, given by

$$\overbrace{(N\pi/12)\begin{bmatrix}(16+15\cos\theta_s+\cos 3\theta_s)\omega_1\\(16+15\cos\theta_s+\cos 3\theta_s)\omega_2\\32\cos(\theta_s/2)^4(2-\cos\theta_s)\omega_3\end{bmatrix}}^{\text{drag with basement membrane}} + \overbrace{(N\mu\sin(\theta_s)^2\pi/2)\begin{bmatrix}2\cos(\theta_s)^2\omega_1\\2\cos(\theta_s)^2\omega_2\\\sin(\theta_s)^2\omega_3\end{bmatrix}}^{\text{drag with pre-stalk}} =$$
$$\underbrace{\sum_{i=1}^{N}\mathbf{r}^i\times\mathbf{b}^i}_{\text{active crawling}} + \underbrace{(NK\pi\sin(\theta_s)^2/8)\begin{bmatrix}A_{32}(-4+3A_{33}+(-4+5A_{33})\cos 2\theta_s)\\A_{31}(4-3A_{33}+(4-5A_{33})\cos 2\theta_s)\\0\end{bmatrix}}_{\text{elastic response of pre-stalk}}, \tag{S26}$$

bioRxiv preprint doi: https://doi.org/10.1101/2025.09.03.674060; this version posted September 4, 2025. The copyright holder for this preprint (which was not certified by peer review) is the author/funder, who has granted bioRxiv a license to display the preprint in perpetuity. It is made available under aCC-BY 4.0 International license.

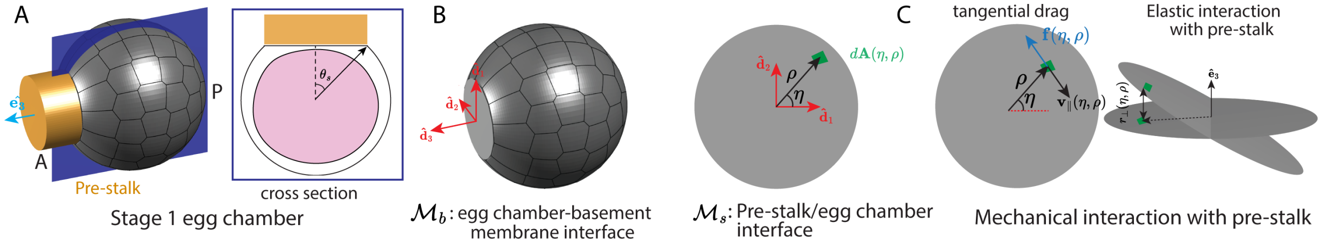

Figure S6: **Model of mechanical interactions in a stage 1 egg chamber.** (a) Geometry of a stage 1 egg chamber. (b) The pre-stalk/egg chamber interface $\mathcal{M}_s$ is parameterized by the polar coordinate (η, ρ). (c) Motion tangential (normal) to the interface is assumed to have a viscous (elastic) resisting force.

where $\mu = \mu_s/\mu_b$ and $K = k_s/\mu_b\omega_c$. To simulate these equations, we use the same parameter values for τ_{1-3} as the delayed migration egg chamber, but with $N = 100$ corresponding to a stage 1 egg chamber. There are no experimental measurements of either μ or K; therefore, we set $\mu = 1$ and $K = 20$. We observe no significant change in the qualitative behavior of the model even for significant changes in these parameter values, consistently generating egg chamber rotations along the pre-stalk axis at steady state.

References

1. Taylor, J. R. *Classical mechanics* (University science book, 2005).
2. Cetera, M. *et al.* Epithelial rotation promotes the global alignment of contractile actin bundles during Drosophila egg chamber elongation. *Nature Communications* **5,** 5511 (2014).
3. Yamaguchi, N. *et al.* Rear traction forces drive adherent tissue migration in vivo. *Nature Cell Biology* **24,** 194–204 (2022).
4. Crest, J., Diz-Muñoz, A., Chen, D.-Y., Fletcher, D. A. & Bilder, D. Organ sculpting by patterned extracellular matrix stiffness. *eLife* **6,** e24958 (2017).
5. Töpfer, U., Guerra Santillán, K. Y., Fischer-Friedrich, E. & Dahmann, C. Distinct contributions of ECM proteins to basement membrane mechanical properties in Drosophila. *Development* **149,** dev200456 (2022).
6. Chlasta, J. *et al.* Variations in basement membrane mechanics are linked to epithelial morphogenesis. *Development* **144,** 4350–4362 (2017).
7. Chen, D.-Y., Crest, J., Streichan, S. J. & Bilder, D. Extracellular matrix stiffness cues junctional remodeling for 3D tissue elongation. *Nature Communications* **10,** 3339 (2019).
8. Haigo, S. L. & Bilder, D. Global tissue revolutions in a morphogenetic movement controlling elongation. *Science* **331,** 1071–1074 (2011).
9. Barlan, K., Cetera, M. & Horne-Badovinac, S. Fat2 and Lar Define a Basally Localized Planar Signaling System Controlling Collective Cell Migration. *Developmental Cell* **40,** 467–477.e5 (2017).
10. Williams, A. M., Donoughe, S., Munro, E. & Horne-Badovinac, S. Fat2 polarizes the WAVE complex in trans to align cell protrusions for collective migration. *eLife* **11,** e78343 (2022).

bioRxiv preprint doi: https://doi.org/10.1101/2025.09.03.674060; this version posted September 4, 2025. The copyright holder for this preprint (which was not certified by peer review) is the author/funder, who has granted bioRxiv a license to display the preprint in perpetuity. It is made available under aCC-BY 4.0 International license.

Movie legends

Movie 1. Egg chamber rotation occurs within the germarium Related to Figure 1. An example egg chamber transitioning from stage 1 to stage 2, which is signified by the basement membrane (Col-IV-GFP) becoming continuous over the anterior portion of the egg chamber. This egg chamber undergoes sustained rotations for the length of the movie. This sample was used to generate Fig. 1E,F. Nuclei were visualized using SpyDNA, which labels all follicle cell and germ cell nuclei. Time stamp shows hours:minutes.

Movie 2. Egg chamber rotation initiates during stage 1. Related to Figure 1. The follicular epithelium of two different stage 1 egg chambers undergoing either sustained rotation (left) or initiating rotation (right). The yellow arrow appears at the onset of rotation. These egg chambers were used to generate the kymographs in Fig. 1I. Nuclei were visualized using SpyDNA, which labels all follicle cell and germ cell nuclei. Time stamp shows hours:minutes.

Movie 3. *109-30>fat2 RNAi* delays the onset of migration. Related to Figure 2. A control ovariole (left) and *109-30>fat2* RNAi ovariole (right) each containing a stage 3 and stage 6 egg chamber, focused on the follicular epithelium. Temporal knock down of Fat2 using the 109-30-Gal4 driver (right) blocks migration specifically in the stage 3 egg chamber, while migration occurs normally in the adjacent stage 6 egg chamber. Follicle cells in both stage 3 and 6 egg chambers migrate in the control ovariole (left). Cell membranes were visualized using CellMask.

Movie 4. Rotation can initiate *ex vivo*, after a delay in *fat2* expression. Related to Figure 2. The follicular epithelium of (left) two control egg chambers undergoing continuous rotation *ex vivo*, and (right) four examples of *109-30>fat2 RNAi* egg chambers undergoing delayed onset of rotation *ex vivo*. All examples are of stage 5 egg chambers. Indy-GFP labels follicle cell membranes. Time stamp shows hours:minutes.

Movie 5. Tracking of cell centroid positions. Related to Figure 4. The basal surface of a *109-30>fat2 RNAi* epithelium in a stage 5 egg chamber pre-rotation to demonstrate how cell centroid positions are used to track cell movement. Cell membranes were visualized using CellMask.

Movie 6. Follicle cells' basal surfaces are highly dynamic before rotation begins. Related to Figure 4. Example of a *109-30>fat2 RNAi* epithelium that is rotating, a *109-30>fat2 RNAi* epithelium pre-rotation, and a $fat2^{N103\text{-}2}$ epithelium that will never rotate. In rotating egg chambers, the apical and basal surfaces move in concert, while motion in pre-rotating egg chambers is restricted to the basal epithelial surface. This local motility is dependent on Fat2 activity. All examples are of stage 5 egg chambers. Samples were used to generate Fig. 4A. Cell membranes were visualized using CellMask.

Movie 7. Simulation of the delayed onset of egg chamber rotation. Related to Figure 5, panel F. The time evolution of the biophysical model of a delayed migration egg chamber with an ellipsoidal geometry.

Movie 8. Anterior-most follicle cells migrate past pre-stalk cells in stage 1 egg chambers. Related to Figure 6. Timelapse of a stage 1 egg chamber with pre-stalk cells highlighted in orange and anterior-most follicle cell in magenta. Over time, the anterior-most follicle cells slide past the pre-stalk cells which remain stationary. This sample was used to generate Fig. 6B. Nuclei visualized with ubi-mRFP-NLS. Time stamp shows hours:minutes.

Movie 9. Simulation of the onset of rotation at stage 1. Related to figure 6, panels F and G. The time evolution of the biophysical model of a stage 1 egg chamber.

Movie 10. Simulation of spherical egg chamber rotation. Related to figure S5. The time evolution of the biophysical model of a delayed migration egg chamber with a spherical geometry.

bioRxiv preprint doi: https://doi.org/10.1101/2025.09.03.674060; this version posted September 4, 2025. The copyright holder for this preprint (which was not certified by peer review) is the author/funder, who has granted bioRxiv a license to display the preprint in perpetuity. It is made available under aCC-BY 4.0 International license.

Table 1: Resources and reagents

**Reagent type (species) or resource**	**Designation**	**Source**	**Identifiers**
antibody	anti-DN Cadherin	Developmental Studies Hybridoma Bank	Antibody Registry ID: AB_528121
antibody	Alexa Fluor 647, goat anti-rat secondary	Thermo Fisher Scientific	Cat# A21450
chemical compound, drug	CellMask™ Deep Red Membrane Stain	Thermo Fisher Scientific	Cat# C10046
chemical compound, drug	SPY650-DNA	Cytoskeleton, Inc.	Cat# CY-SC501
chemical compound, drug	Alexa Fluor™ 647 phalloidin	Thermo Fisher Scientific	Cat# A22287
chemical compound, drug	Invitrogen™ Rhodamine Phalloidin	Thermo Fisher Scientific	Cat# R415
chemical compound, drug	Formaldehyde, 16%, methanol free, Ultra Pure	Polysciences, Inc.	Cat# NC1040701
chemical compound, drug	Recombinant Human Insulin	Millipore Sigma	Cat# 12643
chemical compound, drug	Schneider's Drosophila Medium	Thermo Fisher Scientific	Cat# 21720024
chemical compound, drug	Fetal Bovine Serum	Gibco™	Cat# A5670701
chemical compound, drug	Soda Lime Solid Glass Microspheres	Cospheric	S-SLGMS-2.5 (various sizes, see methods)
chemical compound, drug	Fibrinogen, Bovine Plasma	Millipore Sigma	Cat# 341573
chemical compound, drug	Thrombin Protease	Cytiva	Cat# 27084601
chemical compound, drug	SlowFade Antifade Kit	Thermo Fisher Scientific	Cat# S36963
chemical compound, drug	PBS, Phosphate Buffered Saline, 10X Solution, Fisher BioReagents™	Thermo Fisher Scientific	Cat# BP399-1
genetic reagent *(Drosophila melanogaster)*	*w[1118]*	Bloomington Drosophila Stock Center	BDSC: 3605; FlyBase ID: FBst0003605
genetic reagent *(Drosophila melanogaster)*	*traffic jam-Gal4 (tj-Gal4)*	Kyoto Stock Center	DGRC: 104055; FlyBase ID: FBst0302922

bioRxiv preprint doi: https://doi.org/10.1101/2025.09.03.674060; this version posted September 4, 2025. The copyright holder for this preprint (which was not certified by peer review) is the author/funder, who has granted bioRxiv a license to display the preprint in perpetuity. It is made available under aCC-BY 4.0 International license.

genetic reagent *(Drosophila melanogaster)*	*109-30-Gal4*	Bloomington Drosophila Stock Center	BDSC: 7023; FlyBase ID: FBti0027548
genetic reagent *(Drosophila melanogaster)*	*UAS-fat2-RNAi*	Bloomington Drosophila Stock Center	BDSC: 40888; FlyBase ID: FBst0040888
genetic reagent *(Drosophila melanogaster)*	*UAS-Abi-RNAi*	National Institute of Genetics, Japan	NIG: 9749R-3; FlyBase ID: FBtp0079430
genetic reagent *(Drosophila melanogaster)*	*20XUAS-6XmCherry-HA*	Bloomington Drosophila Stock Center	BDSC: 97863
genetic reagent *(Drosophila melanogaster)*	*Fat2-3xeGFP,FRT80B*	Lab of S. Horne-Badovinac ; PMID:28292425	FlyBase ID: FBal0326664
genetic reagent *(Drosophila melanogaster)*	*viking-gfp (vkg-GFP)*	Buszczak et al.; PMID:17194782; Morin et al.; PMID:11742088	FlyBase ID: FBal0211825
genetic reagent *(Drosophila melanogaster)*	*indy-GFP*	Bloomington Drosophila Stock Center	BDSC: 50860; FlyBase ID: FBal0212048
genetic reagent *(Drosophila melanogaster)*	*yw; ubi-nls-mRFP, vkg-GFP,FRT40*	Lab of David Bilder; PMID: 21212324	
genetic reagent *(Drosophila melanogaster)*	*fat2$^{N103\text{-}2}$,FRT80B*	Lab of S. Horne-Badovinac ; PMID:22413091	FlyBase ID: FBal0267777
software, algorithm	Fiji (ImageJ)	PMID:22743772; 26153368	
software, algorithm	Zen Blue	Zeiss	
software, algorithm	Zen Black	Zeiss	
software, algorithm	Prism9 GraphPad	GraphPad Software	
software, algorithm	Python3	Python Software Foundation	https://www.python.org
software, algorithm	imageio	imageio contributors	
software, algorithm	napari		https://napari.org/stable/
software, algorithm	numpy		https://numpy.org/

bioRxiv preprint doi: https://doi.org/10.1101/2025.09.03.674060; this version posted September 4, 2025. The copyright holder for this preprint (which was not certified by peer review) is the author/funder, who has granted bioRxiv a license to display the preprint in perpetuity. It is made available under aCC-BY 4.0 International license.

software, algorithm	scipy		https://scipy.org/
software, algorithm	scikit-image		https://scikit-image.org/
software, algorithm	pandas		https://pandas.pydata.org/
software, algorithm	cellpose	Carson Stringer and Marius Pachitariu; PMID: 33318659	https://www.cellpose.org/
software, algorithm	Numbers spreadsheets	Apple Inc.	
software, algorithm	Adobe Illustrator	Adobe	
software, algorithm	MATLAB R2023a	MathWorks	

bioRxiv preprint doi: https://doi.org/10.1101/2025.09.03.674060; this version posted September 4, 2025. The copyright holder for this preprint (which was not certified by peer review) is the author/funder, who has granted bioRxiv a license to display the preprint in perpetuity. It is made available under aCC-BY 4.0 International license.

Table 2: Experimental genotypes

Figure	Panel	Name or label	Genotype
Figure 1	E,F,H,I	Col IV-GFP	*w; vkg-GFP/+*
	G,H		*yw; ubi-nls-mRFP, vkg-GFP,FRT40/+*
Figure 2	A,B	Control	*w; 109-30-Gal4/+; Fat2-3xeGFP,FRT80B*
		109-30>fat2-RNAi	*w; 109-30-Gal4/fat2-RNAi; Fat2-3xeGFP,FRT80B*
		tj>fat2-RNAi	*w; tj-Gal4/UAS-fat2-RNAi; Fat2-3xeGFP,FRT80B*
	C,D	Control	*w; 109-30-Gal4/+*
		109-30>fat2-RNAi	*w; 109-30-Gal4/UAS-fat2-RNAi*
		tj>fat2-RNAi	*w; tj-Gal4/UAS-fat2-RNAi*
	E	Control, cell membranes	*w;; indy-GFP*
		109-30>fat2-RNAi, cell membranes	*w; 109-30-Gal4/UAS-fat2-RNAi; indy-GFP*
Figure 3	A-B	Control, Fat2-3xGFP	*w; 109-30-Gal4/+; Fat2-3xeGFP,FRT80B*
		109-30>Abi-RNAi, Fat2-3xGFP	*w; 109-30-Gal4/+; UAS-Abi-RNAi, Fat2-3xeGFP,FRT80B/Fat2-3xeGFP,FRT80B*
		tj>Abi-RNAi, Fat2-3xGFP	*w; tj-Gal4/+; UAS-Abi-RNAi, Fat2-3xeGFP,FRT80/Fat2-3xeGFP,FRT80*
Figure 4	A,B,D,E	*109-30>fat2-RNAi* (Rotating and pre-rotation)	*w; 109-30-Gal4/UAS-fat2-RNAi; Fat2-3xeGFP,FRT80B*
		$fat2^{N103-2}$ (Will never rotate)	*w;; $fat2^{N103-2}$,FRT80B*
Figure 6	B	Nuclei	*yw; ubi-nls-mRFP, vkg-GFP,FRT40/+*
S1	A-C	Col-IV-GFP	*w; vkg-GFP/+*
	D	*tj>UAS-mCherry*	*tj-Gal4/+; 20xUAS-6xmCherry-HA*
		109-30>UAS-mCherry	*109-30-Gal4/+; 20xUAS-6xmCherry-HA*
	E	Control, Fat2-3xGFP	*w; 109-30-Gal4/+; Fat2-3xeGFP,FRT80B*
		10930>fat2-RNAi, Fat2-3xGFP	*w; 109-30-Gal4/UAS-fat2-RNAi; Fat2-3xeGFP,FRT80B*
	F	Control	*w; 109-30-Gal4/+*
		109-30>Abi-RNAi	*w; 109-30-Gal4/+; UAS-Abi-RNAi/+*
		tj>Abi-RNAi	*w; tj-Gal4/+; UAS-Abi-RNAi/+*
S2	B,C	*109-30>fat2-RNAi* (Rotating and pre-rotation)	*w; 109-30-Gal4/UAS-fat2-RNAi; Fat2-3xeGFP,FRT80B*
		$fat2^{N103-2}$ (Will never rotate)	*w;; $fat2^{N103-2}$,FRT80B*
	D,E	Nuclei	*yw; ubi-nls-mRFP, vkg-GFP,FRT40/+*
